# Applying Multiple Methods to Comprehensively Evaluate a Patient Portal’s Effectiveness to Convey Information to Patients

**DOI:** 10.2196/jmir.5451

**Published:** 2016-05-17

**Authors:** Jordan M Alpert, Alex H Krist, Rebecca A Aycock, Gary L Kreps

**Affiliations:** ^1^ Department of Health Behavior and Policy Virginia Commonwealth University Richmond, VA United States; ^2^ Department of Family Medicine and Population Health Virginia Commonwealth University Richmond, VA United States; ^3^ Department of Communication George Mason University Fairfax, VA United States

**Keywords:** eHealth, patient portal, health communication, qualitative study, case study

## Abstract

**Background:**

Patient portals have yet to achieve their full potential for enhancing health communication and improving health outcomes. Although the Patient Protection and Affordable Care Act in the United States mandates the utilization of patient portals, and usage continues to rise, their impact has not been as profound as anticipated.

**Objective:**

The objective of our case study was to evaluate how well portals convey information to patients. To demonstrate how multiple methodologies could be used to evaluate and improve the design of patient-centered portals, we conducted an in-depth evaluation of an exemplar patient-centered portal designed to promote preventive care to consumers.

**Methods:**

We used 31 critical incident patient interviews, 2 clinician focus groups, and a thematic content analysis to understand patients’ and clinicians’ perspectives, as well as theoretical understandings of the portal’s use.

**Results:**

We gathered over 140 critical incidents, 71.8% (102/142) negative and 28.2% (40/142) positive. Positive incident categories were (1) instant medical information access, (2) clear health information, and (3) patient vigilance. Negative incident categories were (1) standardized content, (2) desire for direct communication, (3) website functionality, and (4) difficulty interpreting laboratory data. Thematic analysis of the portal’s immediacy resulted in high scores in the attributes enhances understanding (18/23, 78%), personalization (18/24, 75%), and motivates behavior (17/24, 71%), but low levels of interactivity (7/24, 29%) and engagement (2/24, 8%). Two overarching themes emerged to guide portal refinements: (1) communication can be improved with directness and interactivity and (2) perceived personalization must be greater to engage patients.

**Conclusions:**

Results suggest that simple modifications, such as increased interactivity and personalized messages, can make portals customized, robust, easily accessible, and trusted information sources.

## Introduction

There is significant progress to be made in creating digital communication platforms that improve health outcomes [1]. While patient portals have been lauded as a method to enhance health communication [2], and they have been found to be helpful, their impact has not been as profound as anticipated. Yet patients are still enthusiastic about the portals’ capabilities to assist in managing their health [3]. In one study, 43% of patients believed that apps, such as portals, could improve relationships with doctors, 48% would feel more in control of their health, and 40% would be would encouraged to ask physicians more questions [4]. Evidence has demonstrated that portals contribute to improved health outcomes by increasing cancer screenings, especially when information was explained in lay language, used personalized recommendations, and provided educational resources [5]. Still, clinicians report dissatisfaction [6] and patients are underwhelmed with the design and functionality of portals [7].

Incorporating immediacy into portal design has the ability to increase their usability and importance as health communication resources. Immediacy refers to features that promote physical and emotional closeness, caring relationships, authenticity, and enthusiasm [8]. The construct has a rich tradition focusing on face-to-face communication in education [9,10], psychological counseling [11,12], and health care delivery [13,14]. However, immediacy has only recently been applied to digital health communication. Incorporating immediacy into the design of health communication tools can better engage, involve, and motivate patients to promote their health and well-being [15]. However, thus far there has been little evidence to demonstrate that portals can create a sense of immediacy [16].

Our study explored how a unique set of methods—critical incident reports from patients, focus groups of clinicians, and thematic analysis—can be used to evaluate and better inform the design of patient portals. We analyzed one exemplar patient portal, MyPreventiveCare [17], as a test of concept. MyPreventiveCare, a highly functional, prevention-focused online resource, was designed specifically to activate and engage patients around preventive care [18]. It is used by 12 practices in Virginia in the United States and reaches over 82,000 patients, but is being extended to an additional 300 practices in 15 states through a series of grants. In addition to providing laboratory results and viewing information from the medical record, the innovative portal customizes content based on hundreds of demographic, historical, behavioral, and clinical variables to make personalized recommendations and provide educational material based on current guidelines using content from HealthFinder.gov, a credible online health information resource from the National Health Information Center (Washington, DC). The portal provides information about needed cancer screenings, how to better monitor chronic conditions, and how to improve health behaviors. Nearly half (43.4%) of patients registered on MyPreventiveCare have logged in to the portal in the past year and frequently visit the following areas: laboratory results, medical record information, and preventive care recommendations. The average patient accesses the portal almost 4 times per year.

## Methods

To sufficiently assess the test portal, we used multiple methods to collect and analyze data. Using multiple methodologies in health science research helps researchers view problems from multiple perspectives to enrich the meaning of a singular viewpoint [19] and contributes to developing a more complete understanding of a problem [20]. We describe the methodology for conducting interviews, focus groups, and the thematic analysis.

### Interviews

#### Sample

Age demographics for current users of the portal are as follows: 18–24 years (3980/72,362, 5.50%), 25–34 (13,387/72,362, 18.50%), 35–44 (16,281/72,362, 22.50%), 45–54 (16,643/72,362, 23.00%), 55–64 (12,302/72,362, 17.00%) and ≥65 (9769/72,362, 13.50%). More females (41,246/72,362, 57.00%) use the system than males (31,116/72,362, 43.00%) and in fact, the most frequent users are women 45–54 years old. Although women 45–54 years old is the largest demographic group of users, criteria for interviews included patients ranging from 18–79 years, to generate as many viewpoints as possible.

We used naturalistic intercept sampling techniques [21], such as purposeful sampling [22] and convenience sampling [21], to recruit active users, age 18-79 years, who accessed the system at least once in the past year. To enhance recruitment, efforts were made to make participation in the interviews convenient for patients who already were at the clinic waiting for their doctors’ appointments. Patients who met eligibility and had an upcoming appointment were contacted via telephone 1 week in advance to schedule an interview before the appointment with their physician. In addition, the medical staff identified eligible patients and referred them either before or after the appointment with their physician. We recruited participants in the fall of 2014 and winter of 2015 in 2 primary care practices that belong to the Virginia Ambulatory Care Outcomes Research Network. The study was described to patients in a private conference room, and then informed consent was reviewed. Scheduling interviews with patients around impending appointments over the course of several weeks allowed for a diverse cross-section of patients to be recruited.

Critical incident technique (CIT) [23] was the main method of inquiry, which is a qualitative research method with strong exploratory and investigative abilities [24-26]. The technique was designed as a flexible set of principles with the goal of identifying incidents that the users considered positive or negative and to probe participants about their experiences. The CIT is especially advantageous to the evaluation of a patient portal because it allows users to reflect on the most meaningful events from their experience using the system. Unlike usability studies, which solely focus on the way an individual operates a system, CIT allows both patients and clinicians to identify instances in which the portal affected their lives outside of directly using the system. CIT has been used to analyze quality of care [25] and is a common method in the application of health care services [27].

#### Procedures

During interviews, participants were asked to recall their best and worst experiences using the portal. These 2 broad questions framed the discussion, allowing the interviewer to focus on the incidents mentioned and investigate their significance. Interviews ceased and saturation was achieved when keywords and phrases were frequently repeated, participants’ discourse was similar, and recurrent meanings were discovered [28]. All discussions were audio recorded and subsequently transcribed. The average interview time was 14 minutes in length.

#### Analytical Process

To ensure objectivity of the incidents collected, we developed a set of specific rules that specified whether an incident was positive or negative based on (1) the situation of the incident, (2) relevance to the general aims of the study, and (3) importance of the incident’s effect on the aims [23]. Data analysis was guided by the tradition of CIT and the constant comparative method [29], in which 2 researchers (JA, GK) independently read each transcript, exploring for prevalent themes. Initially, any incident that could be considered either positive or negative was collected. Upon further analysis, the researchers discussed examples of positive and negative incidents, thereby refining the qualifications to be considered an incident worthy of inclusion. Next, the researchers shared observed themes and formed categories by creating an aggregated codebook. If discrepancies occurred, the researchers referred to the rules and aims of the study and discussed their perspective until consensus was met. Each researcher once again independently analyzed the data and assigned a code that best captured the significance of the incident. The researchers met to review their findings frequently and discussed any discrepancies that arose until they reached a consensus around a particular theme. To assess the validity of the data, we initiated respondent validation, or member checks [30], with several participants to confirm the interpretations of the findings.

### Focus Groups

#### Sample

We conducted 2 focus groups with clinicians in 1 Northern Virginia medical office. The administrator of the portal emailed providers working in the medical office and made an announcement at a monthly meeting about participation in the study. Participation was voluntary and scheduled during providers’ lunch hour. The first focus group included 8 physicians (2 men and 6 women), comprising 4 residents and 4 full-time physicians. The second focus group consisted of 5 participants (4 women and 1 man), 4 nurses and 1 emergency medical technician. The average length of both focus group sessions was 51 minutes.

#### Procedures

We used the CIT to allow clinicians to concentrate on the extreme positive and negative functions of the portal. A printed list of general questions was presented at the beginning of the focus group session. Questions were grouped into the following domains: positive experiences, negative experiences, and how the portal positively or adversely affected the communication process with patients and staff. The questions were semistructured, to allow for organic, flexible conversations that stimulated discussion [22]. Each focus group session was audio recorded and transcribed. For transcripts involving the focus group data, we used the same analytical process previously described for interviews.

### Thematic Analysis of the Portal

We conducted the third method, thematic analysis, after interviews and focus groups were completed. We used patients’ and clinicians’ perspectives as a lens to analyze the content found on the portal, as well as by using key factors of immediacy identified in previous research [15,31,32]: user engagement, personalization, interactivity, enhances understanding, and motivates behavior change (definitions can be found in Table 1 [33-40]). We conducted the thematic analysis by reviewing the portal, searching for specific patterns related to immediacy. To strengthen claims of the thematic analysis, we incorporated numerical results, which enabled the amount of evidence in the data to be quantified to support the conclusions [41].

**Table 1:** Immediacy definitions for thematic analysis of the patient portal MyPreventiveCare

Immediacy feature	Definition
Interactivity	Definitions of interactivity typically focus on 2 measures: (1) 2-way flow of information and (2) rapid exchange of information. Other definitions include control as the main component of interactivity [33], meaning that participants should be able to exercise control over the communication exchange. For the purposes of this study, interactivity includes all 3 components and is considered to be reciprocal and synchronous communication that offers active control [34].
Personalization	According to e-commerce websites, personalization is “the adjustment and modification of all aspects of a website that are displayed to a user in order to match users’ needs and wants” [35]. Although the portal is not an e-commerce website, the previous definition was thoroughly explicated and is relevant to this study.
Engagement	Combining definitions from the fields of marketing [36], media [37], and psychology [38], engagement is considered the level of an individual’s physical, cognitive, and emotional involvement or connection with a specific medium.
Motivates behavior	Motivators are triggers, prompts, cues, or calls to action that encourage a user to take action [39].
Enhances understanding	Factors that enhance understanding, similar to relational understanding, in which an individual knows both what to do and why [40].

### Analytical Process

The first author (JA) logged in to the portal as a test user and analyzed 27 separate content pages, of which 24 were evaluated. The home page, “Dashboard,” and “Library” pages were not analyzed because they only functioned as navigational pages. Every webpage was examined individually to determine the prevalence of each immediacy characteristic. A checklist with each immediacy feature’s definition was designed and individual webpages were meticulously scrutinized using the checklist. After an initial review, each page was checked a second time to confirm initial findings. After the first author conducted the analysis, each of the authors gave input in regard to the identification of themes. In particular, the portal’s administrator reviewed the findings and provided feedback on actual patient and provider experiences. The first author then reevaluated each individual webpage taking into account the administrator’s perspective, as well as the theoretical framework of immediacy features.

The use of multiple methods (interviews, focus groups, and thematic analysis) and recruitment of key stakeholders (patients and clinicians) provided triangulation by using methods with different strengths and limitations to support a single conclusion [42].

This study was given full institutional review board approval by the George Mason University Office of Research Integrity & Assurance. Participants signed an informed consent document and, to ensure confidentiality, audio files were only accessed by the researcher, and personal information, such as names, were de-identified upon transcription.

## Results

The study included 44 total participants in 31 patient interviews (18 women and 13 men) and 2 focus groups (13 clinicians). We collected a total of 142 incidents, 102 negative and 40 positive.

### Patient Interviews

Incidents classified as positive (31/113, 27.4%) were outweighed by negative incidents (82/113, 72.6%). The following themes were most salient throughout positive and negative incidents cited by patients.

#### Positive Incidents

Patient interviews revealed 3 main categories of the portal’s usefulness: (1) the ability to instantly access medical information, (2) availability of clear health information, and (3) patient vigilance.

##### Instant Access

Nearly half of all positive incidents were associated with the instantaneous retrieval of medical information. For example, patient #1, a busy mother, anxiously awaited test results but was unable to call the office during business hours. She appreciated how she could log in to the portal in the evening and look up the results.

Similarly, most patients complained that obtaining laboratory results was a chore involving endless telephone calls, but the portal streamlined the once-laborious process. Patient #8 described how he used to get results before the portal was available. He said, “The office doesn’t have time to call you, or they call, and they don’t get you and leave a message, and you call back and leave a message.”

##### Clear Health Information

Not only did patients appreciate the ability to get laboratory results, but also the portal’s design made it easy to navigate. Upon logging in, patients were presented with a dashboard featuring large, colorful icons used as the website’s primary navigation. Referring to the portal’s interface, patient #30 commented, “It is very icon-driven and it’s all very intuitive, so I don’t find it complicated to use.” In addition, throughout every section of the website, a dictionary is available to look up challenging terminology.

##### Patient Vigilance

The ease of the portal’s navigation contributed to patients being able to carefully monitor their health. Patients used information found on the portal to gauge their health status. Patient #4 monitored her laboratory results to determine whether “medications were working or if there was a problem somewhere.” Correspondingly, patient #11 always checked laboratory results as soon as they were available and said, “If my labs are getting worse, or even if they’re normal, I can make adjustments and monitor it closer.” The availability of health information, such as laboratory results, allowed patients to check differences from previous tests, while prevention recommendations, like getting the flu shot, reinforced efficacious behavior and served as a reminder.

#### Negative Incidents

Negative incidents were classified into 4 main categories: (1) standardized content, (2) the desire for clinicians to communicate directly with the patient, (3) website functionality, and (4) difficulty interpreting laboratory data.

##### Standardized Content

Many patients were unaware that content was personalized based on their electronic medical record and self-reported data. For instance, patient #3 said, “it felt generic. I don’t know if it was specific to my medical history, but it didn’t feel personal.” Patient #12 agreed and said, “I’d want something more focused on me as opposed to something generic like this.”

##### Desire for Direct Communication

After reading the portal’s content, many patients craved information directly from the clinician. For instance, patient #15 wondered, “Can the doctor and the medical provider put what they would propose as next steps?” Although the content that appeared was generated from updates the clinician made to the patient’s record, the language used did not reflect that the content was personally delivered by a clinician. This sentiment was best expressed by a retired woman, patient #10, who was inspired with new questions and concerns after viewing content from the portal. She often consulted advice nurses, but since they were no longer available, she yearned for the portal to be a substitute.

In addition to the desire to interact directly with clinicians, many patients wanted a resource that would be beneficial between scheduled office visits. Patient #4 stated, “once in a while something goofy will happen, so it would be nice to come in here (portal) and read the things that I need.” Similarly, patient #2’s blood pressure often wildly fluctuates and during those times, she thought, “it would be nice if I could say, is this something I should worry about?”

##### Website Functionality

The portal was susceptible to problems experienced by many websites, such as issues logging in and server crashes. Indeed, 20% (16/82) of all negative patient incidents involved an instance in which the portal did not function correctly. Patient #12 remembered a time when he had difficulty recovering his password. He said, “I couldn’t remember what my password was. Even after I got the password, when I went to log in again, it wouldn’t let me.”

When patients were able to log in, data entry mistakes or database errors sometimes caused the portal to inaccurately report results. Patient #5 said, “It’s been hit or miss as far as having the information being correct.” Also, information was not updated to appropriately reflect their health status. Patient #28 cited, “I kept getting these flash things that said I was overdue on a pap exam. I had just been in in April, but it kept flashing at me like I was a bad girl.”

##### Laboratory Data Interpretation is Difficult

Patients appreciated the portal’s preventive care messages, such as a recommendation to get a mammography, but experienced difficulty interpreting diagnostic laboratory information. This accounted for 11% (9/82) of negative incidents. For instance, patient #15 referred to the “Watch Your Weight” page and said, “One question I have is this BMI [body mass index]. Is it just sort of a number pulled out of the air or is it in fact appropriately calculated?” He wanted to better understand what the number actually meant to “take the number seriously.” Similarly, some patients had trouble deciphering the results of the blood sugar section, as exemplified by patient #27, who examined the chart on the webpage and wondered, “It was measured on September 3rd. I have a value of 100 and my goal is to be less than 126, so I should be good? I don’t know what that symbol means. [Orange icon indicating a marginal score]. Why would I be marginal?”

### Clinician Focus Groups

We combined data from the 2 focus groups, one with physicians and another with rooming staff. Clinician incidents were comparable with patient data, with 69% (20/29) negative and 31% (9/29) positive.

#### Positive Incidents

Positive incidents among clinicians were classified into 2 main areas: (1) patients feel empowered (5/9, 56%) and (2) the portal can generate office efficiency (4/9, 44%).

##### Develops Patient Empowerment

Over half of all positive incidents involved the belief that patient access to medical information was beneficial. A nurse recalled, “I work with patients who have jobs that take them out of the country and the fact that they have this information is awesome.” Before the introduction of the portal, nurses would review laboratory results over the phone, but patients did not have access to the data as a reference. Furthermore, clinicians observed higher levels of patient motivation after interacting with the portal. A nurse said, “[Patients] will use the portal to track things, like ‘my cholesterol wasn’t that good, so I need to increase my exercise’, and next time, they’ll come in and say, ‘I added another day to my exercise routine and I’m really anxious to see how my cholesterol is now.’”

Due to increased levels of motivation, patients experienced more productive office visits. A physician noticed that the portal “helps start a conversation when there’s so much to cover.” Another physician confirmed that notion and said the portal created “patient led agendas rather than the physician leading the agenda.” He continued, “I’ve had patients who have already looked at their laboratory results before they come in.” Physicians noticed that patients altered their behavior and even researched possible treatment options before their appointment.

##### Generates Workplace Efficiencies

Although many more negative incidents focused on how the portal has the potential to create additional work, positive incidents highlighted how the portal contributes to a more efficient working environment. For instance, reminders delivered to patients via the portal lessened the need for unit clerks to call and remind patients about upcoming examinations. A physician agreed that reminders were particularly useful because they prevented unit clerks from interpreting medical information. In addition, less time was devoted to playing “phone tag” because patients could correct errors, such as 1 incident in which a patient’s file stated that her last colonoscopy was 12 years ago, even though the procedure was performed 5 years ago.

#### Negative Incidents

Clinicians’ incidents overlapped with patients’, and in many cases, reinforced what patients experienced. Negative incidents fell into 3 main categories: (1) lack of feedback, (2) fears that the portal can increase workload, and (3) inappropriate use of the system.

##### Lack of Feedback

Clinicians considered the portal a valuable tool, but acknowledged that it contained flaws. Perhaps the biggest flaw, which accounted for 40% (8/20) of negative incidents, was that clinicians could not confirm whether patients viewed or understood information that was input into the portal. A nurse summarized the problem by describing how she typed the analysis of laboratory results or medication directions, but was unaware whether patients either saw the information input or understood its meaning.

In other scenarios, clinicians received inquiries from patients, but the portal was not deemed an appropriate forum to discuss the matter. For example, a resident recalled the following predicament: “You get this message and you want to answer it...but I find that if that question pushes the boundaries of what I should do outside of an office visit, maybe I should bring them in? But they’re asking for it, so I send the anti-biotic I otherwise wouldn’t.”

Another issue that compressed communication between patients and clinicians was the belief that the portal was not equipped to handle complex communication. A doctor brought up the issue of cancer screening and said, “The problem is there are different ways to do it. We all know we should screen for colon cancer. When I see a patient in person, I ask them do you want to do a colonoscopy or annual stool test?” According to clinicians, a much richer communication platform was needed to conduct meaningful conversations.

##### Fears of Increased Workload

Although clinicians cited specific instances in which the portal generated efficiency in the office, there was still speculation that increased use of the portal would create additional work. Nurses were worried that patients would call the office at greater frequencies because sometimes “patients look at laboratory results and one thing is a little off, so they freak out and they’re calling us and asking.” Physicians were also concerned about devoting more time to addressing patients’ questions because, as one doctor said, “It is frankly time we don’t get reimbursed for.” Since physicians did not receive confirmation that the patient viewed the message, they had to take the time to call the patient and ensure the information was communicated.

##### Inappropriate Use of the System

Surprisingly, clinicians acknowledged that they sometimes purposefully contributed complex medical jargon. For instance, nurses said that younger doctors entered sophisticated terms because they wanted to impress their supervisors who may see the information they input. Physicians with more seniority were also at fault. Nurses complained that many doctors wrote messages meant for nurses and were unaware that patients were also capable of viewing them.

Sometimes, physicians recognized that content on the portal was not written in a sensitive manner. A doctor said, “I’ve had a few patients who get the health maintenance reminders and say, ‘my portal says I’m fat.’” Other doctors felt that the content on the portal may be suitable for the average patient, but might be beyond comprehension for patients who are not native English speakers.

##### Thematic Analysis of the Portal

On the basis of attributes of immediacy (interactivity, personalization, engagement, motivates behavior, enhances understanding), we examined the portal as to whether the characteristics were present (summarized in Table 2).

**Table 2:** Presence (+) or absence (–) of immediacy characteristics in the patient portal MyPreventiveCare.

		Enhances understanding	Personalization	Motivates behavior	Interactivity	Engagement
**Preventive care recommendations**						
	Summary page	–	+	+	+	+
	Take aspirin	+	+	+	–	–
	Get tested for diabetes	+	+	+	–	–
	You have high blood pressure	+	+	+	–	–
	Get a tetanus shot	+	+	+	–	–
**Other recommended behavior**						
	Mammogram	+	+	+	+	–
	Cervical cancer	+	+	+	+	–
	Colon cancer testing	+	+	+	+	–
	Cholesterol	+	+	+	–	–
	Diet	+	+	+	–	–
	Exercise	+	+	+	–	–
	Smoking	+	+	–	–	–
	Weight	–	+	–	–	–
	Pneumonia vaccine	+	+	+	–	–
	Flu vaccine	+	+	–	–	–
	Bone density	+	+	+	–	–
**Laboratory results**						
	Your labs	–	+	–	–	–
**Edit information section**						
	Update page	+	+	+	+	+
**Library**						
	Dictionary	–	–	–	–	–
	Setting priorities	–	–	–	–	–
	Prevention topics A–Z	+	–	+	+	–
	Self-management tools	+	–	+	+	–
**Help**						
	Help page	+	–	+	–	–
**Contact us**						
	Contact page	n/a^a^	–	–	–	–
	Characteristics present, n (%)	19/23 (78%)	18/24 (75%)	17/24 (71%)	7/24 (29%)	2/24 (8%)

^a^n/a: not applicable.

##### Enhances Understanding

This category measured whether content allowed patients to understand not only what to do, but why to do it. We did not review the “Contact” page because its content was not applicable. This section received the highest score, with 78% (18/23) of the portal’s content contributing to enhancing understanding.

The way in which information was presented was integral to enhancing patients’ understanding. Patient #28 commented how the bulleted lists made it easier to process recommendations. Moreover, the use of graphics supported the text it accompanied. For example, an image on the “Exercise” page of a man playing basketball contributed to reinforcing healthy behavior, while photographs of individuals eating fresh vegetables on the “Diet” page emphasized the concept of healthy eating and made it more appealing. Showcasing positive behaviors supplemented the information presented and served as a model to apply recommendations. Other factors that enhanced understanding were detailed explanations. For instance, on the “Take aspirin” page, an initial header outlined the patient’s health status, such as whether they smoked and whether they were taking an aspirin dosage. Next, a brief explanation on the benefits of aspirin appeared, followed by the criteria for whether aspirin is appropriate, next steps, and several related links to more information about aspirin, as well as heart disease. Similarly, T-scores were described in the “Bone density” section, including how weight influences blood pressure. Throughout the portal, simple and direct language was used, demonstrated by the average word length at only 6 letters, which contributes to enhancing patient understanding.

##### Personalization

Personalization was determined by an enhanced sense of inclusion and cooperation to match users’ needs and wants. The main way personalization was achieved came through language, such as using pronouns (“we” or “you”), as well as providing unique information applicable to an individual’s life. For instance, recommendations were offered, like “You are due to get another tetanus shot now” and, after listing a patient’s medical information, the following text was generated: “These conditions place you at higher risk for heart disease.” The portal’s customized content contributed to 75% (18/24) of pages having the quality of personalization.

Although we gave credit for personalization, terms like “you” and “your” were inconsistent. On the “Your labs” page, information was written in a very clinical manner, negating past attempts at personalization. The text read, “Patient would like a mammogram,” instead of using “you” or the patient’s name. This was especially true on the dashboard, which was the first page that appeared when a patient logged in. At the top, it read, “You are here,” instead of immediately establishing personalization by using the patient’s name.

Other areas of the portal lacking personalization were the “Dictionary” and “Prevention topics” pages, which listed topics that could be irrelevant to patients, such as information about cervical cancer to male patients. Lastly, stock photographs of physicians were used throughout the portal instead of photos of clinicians familiar to patients.

##### Motivates Behavior

Aspects of the portal that motivated positive health behavior had to be actionable, meaning participation was encouraged to perform the activity, like scheduling an exam. Over 70% (17/24, 71%) of content had features that could motivate behavior. Every page within the “Preventive care you need now” section fulfilled the motivational behavior criteria by using verbs that promoted action, like “talk with your doctor” and “protect yourself.” Understanding that losing weight is challenging, on the “Your next steps” section of the “Diet” page, the text attempted to motivate patients by stating, “Keep eating five or more servings of vegetables and fruits per day.” However, we did not consider the “Smoking,” “Weight,” and “Flu vaccine” pages to be motivational because the content was overly passive. Confirming this analysis, patient #31, with diabetes, thought that the content should be “scarier” to motivate people to change their behavior. She acknowledged that losing weight would assist with her diabetes management, but after reviewing the content, she said, “I’m not scared enough to be doing all the things I ought to be doing.” Similarly, the “Flu” page recommended as a next step, “Talk with your doctor about getting a flu shot.” Although an action verb was used and previous content provided an overview of benefits, no urgency to get the vaccine was generated.

##### Interactivity

Interactivity included synchronous communication, the ability of a user to take control, or any function that enabled the user to become involved more deeply within the content. We found only 29% (7/24) of the pages to have characteristics of interactivity. There were very few opportunities to interact with the portal aside from reading text. Interactive quizzes, cholesterol calculators, and other risk assessment tools were presented as external links, which took the user away from the portal. Lack of interactivity was confirmed by a doctor who was frustrated over the system’s asynchronous communication. He said, “The patient can’t contact us directly. Instead, they have to go through the nurse and we review it a few hours later, or a day later, and then it becomes broken communication.” However, a relatively strong aspect of the portal’s interactivity was the ability for the user to control the navigation and choose the area accessed. For instance, the “Self-management tools” page allowed for patients to find specific information relevant to their condition, while the mammogram and cervical cancer pages enabled the patient to input dates of their last test and whether abnormal results appeared.

Opportunities for greater interactivity were available on most pages. For instance, the “Take aspirin” page asked questions about risk, but there was no way for the patient to answer those questions and receive timely feedback. Also, the “Your labs” page had a row of physician comments, but it was not possible for the patient to respond, make an appointment, or get more information.

##### Engagement

Engagement received the lowest score with 8% (2/24). Only 2 webpages were deemed engaging, having content that made the user want to further explore the website by creating physical, cognitive, or emotional involvement.

The main “Preventive care you need now” page made bold proclamations, like “You have high blood pressure,” which would get a patient’s attention and impel them to further explore. The rest of the pages did not have enough personalized content to be engaging. For example, the “Mammogram” page made a recommendation for women age 40–84 years instead of providing more specific advice based on the patient’s specific age. The “Self-management tools” page included links within 19 specific content categories, including exercise, healthy diet, and smoking cessation, but required that a patient scroll through almost 200 links.

### Consequences of Incidents and Immediacy Levels

We synthesized the patients’ and clinicians’ incidents with results of the content analysis to identify domains that would improve website functionality. We discovered the following 2 themes: (1) communication can be improved with directness and interactivity and (2) standardization contributes to patient disengagement.

### Communication Can Be Improved With Directness and Interactivity

While the intention of the portal was to provide direct information to augment the patient’s care, both patients and clinicians found that the portal sometimes hampered the communication process. The portal provided general awareness overviews; however, patients yearned for specific information about how a particular treatment personally affected them. Therefore, patients used other online sources for more specific information.

Patient #9 said, “Sometimes I have back pain and I cannot come to the primary care doctor, but I need some information.” This patient’s comments aligned with many other patients’ views, whose first instinct was to seek outside sources rather than rely on the portal. Furthermore, patients needed detailed information between visits to manage chronic diseases. For instance, patient #31, with type 2 diabetes, was concerned about managing her blood sugar. She said, “I’m not sure I’m always doing the right things” and the portal could not be counted on as a resource to help her manage specificities of the disease. Patients became disinterested in the portal’s content, because as patient #19 said, “I pretty much know all this stuff.” Patient #21 considered the portal “dumbed down” and mentioned that, since he was due for a prostate exam, he already conducted basic research. Perhaps, if patients deemed content to be authoritative and felt that it was coming directly from the clinician, they would find it more helpful and seek out the portal between scheduled visits. By using alternative websites, patients would combine the vetted and personalized information from the portal with recommendations from the other websites, which may or may not be beneficial given the patient’s specific medical history.

Similarly, clinicians reported that they were unable to effectively communicate in an appropriate manner through the portal. They were unsure whether patients read their instructions, and asynchronous communication patterns disrupted care.

### Standardization Contributes to Patient Disengagement

Although content was personalized, patients largely viewed the portal as just another platform offering standardized information, which minimized patients’ assessment of it as a resource for delivering personalized health recommendations.

#### Lack of Personal Relevance

Patient #25 became indifferent and dismissed the portal as a helpful tool after reading the section about weight loss. He responded, “Yeah, but what do I do with it? I’m overweight. I need to lose weight. Got it.” Patient #8 was unimpressed with the recommendations and said, “A lot of the information, the basics, this is stuff I could read on CNN health’s website. [It] doesn’t tell me much.” Similarly, patient #20 was disappointed with the portal’s lack of guidance about vitamins. Since the efficacy of vitamins is not evidence based and therefore would not be recommended by the clinician, the portal’s content did not address vitamins.

#### Not Motivational

Overall, the portal did not provide actionable information, leaving patients with little motivation to use the system. Patient #27 said, “This is not motivating. Maybe if it gave me examples, like go for a walk or have an apple with lunch.” Lack of specificity was best exemplified by patient #4, who visited the physician for a flu shot even though it had been years since she last received the vaccine. She thought the portal’s flu shot content was “generic” and it did not inspire her, but said, “I am here today because I’ve been told by my daughter’s baby’s pediatrician that I need to get a flu shot.”

## Discussion

### Principal Findings

Conducting a multiple methodological study on a patient portal as a case study revealed that many factors need to be considered when delivering personalized health information via a portal. It was necessary to use multiple methods and involve numerous stakeholders to truly understand the complexity of the user experience. The combination of gathering critical incidents from patients and clinicians, and analyzing the themes of the portal’s content enabled us to simultaneously confirm the results through a concurrent triangulation strategy [43].

Findings suggest that personalized content must contain higher levels of engagement and interactivity. This may be accomplished through increased levels of immediacy, which has been associated with greater patient satisfaction, understanding [13,44], and compliance [45]. Communication strategies incorporating immediacy, like adding personalized content addressing patients’ concerns about treatment, can contribute to humanizing interactions between patients and clinicians, encouraging patient participation and building trust [8].

Making portals more immediate through other forms of health information technology hold the opportunity for greater levels of engagement [46]. For instance, interactive videos to inform patients about elective back surgery helped facilitate decision making and informed consent [47]. Compared with reading text, watching videos has the ability to improve patients’ knowledge of health risks [48]. Furthermore, videos featuring information directly from a patient’s clinician could make the content seem more authoritative and personal.

Although implementation and integration of health portals has initially been slow [49], dramatic growth is expected due to patient demand and physician adoption [50]. Therefore, it is necessary to design patient-centric systems that provide comprehensive, coordinated value to patients, while also meeting the needs of clinicians [51]. In fact, clinician endorsement and engagement with the portal is an important factor that influences patient adoption [52].

The portal used for this case study has been at the forefront of personalizing content, but even more is needed. While the majority of webpages fulfilled the definition of personalization, patients expected a higher level of personalization akin to sophisticated mobile phones and social networking websites. This tension between patients’ expectations of personalization and clinician guidelines is pronounced because patients’ expectations of the portal’s main function differ from clinicians’. While the goal of the portal used in this study was primarily to educate and motivate patients for preventive screenings, patients considered the portal to be a mechanism to organize all of their health care needs. Sections with the highest level of personalization were on the preventive care pages, but patients expected similar personalization levels on laboratory results and medication pages. Based on this study’s findings, the objective of portals needs to be better communicated to patients, and future modifications should consider patients’ expectations. A portal rich with immediacy not only has the capacity to fulfill patients’ desires, but also has the potential to improve the medical environment. Results from this study indicated that clinicians worry about increases in workload, but they also realize the potential for portals to create greater efficiency.

### Limitations and Future Directions

There were several limitations to this study. It would have been beneficial to have greater representation of adults from various age ranges, ethnicities, and socioeconomic classes. Participants represented only a small subset of all users, and recruitment likely selectively targeted those with strongly positive or negative impressions. In addition, comparisons across other health care information systems would have been beneficial to contrast differences and illuminate the various ways that systems are used. Future research should focus on analyzing the content, design, and usability of portals. In addition, research should analyze how patients use the portal’s content to determine whether it is a factor influencing health behavior.

### Conclusions

Through the analysis of this portal, we found that higher levels of immediacy are necessary to sufficiently motivate patients to take preventive care measures. Portals are becoming a fixture of every medical office; therefore, it is necessary to modernize portals so that they can achieve their potential of enhancing health communication and improving health outcomes. The methods used in this study can be replicated when analyzing other patient portals, and conclusions can inform designers to develop more effective health information communication.

## Abbreviations

CIT: critical incident technique
